# An Automated Planning Model for HRI: Use Cases on Social Assistive Robotics

**DOI:** 10.3390/s20226520

**Published:** 2020-11-14

**Authors:** Raquel Fuentetaja, Angel García-Olaya, Javier García, José Carlos González, Fernando Fernández

**Affiliations:** Computer Science Department, Universidad Carlos III de Madrid, 28911 Leganés, Spain; agolaya@inf.uc3m.es (A.G.-O.); fjgpolo@inf.uc3m.es (J.G.); josgonza@inf.uc3m.es (J.C.G.); ffernand@inf.uc3m.es (F.F.)

**Keywords:** Automated Planning, Human-Robot Interaction, Social Assistive Robotics, knowledge representation

## Abstract

Using Automated Planning for the high level control of robotic architectures is becoming very popular thanks mainly to its capability to define the tasks to perform in a declarative way. However, classical planning tasks, even in its basic standard Planning Domain Definition Language (PDDL) format, are still very hard to formalize for non expert engineers when the use case to model is complex. Human Robot Interaction (HRI) is one of those complex environments. This manuscript describes the rationale followed to design a planning model able to control social autonomous robots interacting with humans. It is the result of the authors’ experience in modeling use cases for Social Assistive Robotics (SAR) in two areas related to healthcare: Comprehensive Geriatric Assessment (CGA) and non-contact rehabilitation therapies for patients with physical impairments. In this work a general definition of these two use cases in a unique planning domain is proposed, which favors the management and integration with the software robotic architecture, as well as the addition of new use cases. Results show that the model is able to capture all the relevant aspects of the Human-Robot interaction in those scenarios, allowing the robot to autonomously perform the tasks by using a standard planning-execution architecture.

## 1. Introduction

Social robots [1] must autonomously interact with people on dynamic and uncertain environments. A common way to control such robots is the use of finite-state machines (FSM) [2,3,4,5,6], where each state corresponds to a certain situation during the interaction, and transitions between states depend both on actions performed by the robot and information received by sensors. FSMs are simple and fast mechanisms to control the robot, particularly in structured environments, but, in more sophisticated applications, to identify and correctly specify all possible states that could appear and the transitions among them can be a really hard task. Moreover, adding or modifying functionality once the robot is deployed can be very difficult given that all behaviors are heavily hard-coded.

Automated Planning (AP) allows to model the task using declarative languages, and general problem-solving techniques are used to build plans from the initial state to a state where goals are achieved. AP is a broad field, where various approaches, like Action-based Planning [7] or Timeline-based planning [8], and different representation languages [9,10] coexist. The approach used in this work, Classical Planning, and its extensions belong to the Action-based Planning paradigm. This allows to model the actions the robot can perform and the possible states of the system using a predicate-logic based language, the Planning Domain Definition Language (PDDL) [10]. There are some other works in the robotics literature using either timeline-based planning [8,11,12,13,14], action-based planning [15,16,17], or a mix of them [18,19]. However, only few of them deal with HRI on Social Assistive Robots [14,18,19]. AP has been also applied in the context of HRI for other tasks, as language, dialog and conversation generation [20,21,22,23] and situated natural language generation [24].

Using AP for high level control of robots has several advantages [13,25]. With AP, the model specifies action schemes in terms of the facts that must be true for the action to be applied (preconditions), the changes in the state after the action is executed (effects), the initial state and the goals. Then, a domain-independent planner will find the sequence of actions that once executed allow to reach the goals. Thus, it is not required to enumerate every state and every possible transition, as in FSMs, and additionally any improvement from the planning community can be incorporated just by selecting the best planner available.

However, even in the case of simplest task representation models, like the ones generated for Classical Planning using PDDL2.1 [26], formalizing complex tasks is hard for non-expert engineers [27]. Insufficient knowledge about the rationality of the causal models, the limitations of current planners, or even the lack of good editors make it difficult to use AP solutions. This is the case of the area of Human-Robot Interaction, where specific tasks, like Comprehensive Geriatric Assessment (CGA) or robotic rehabilitation are very complex to model even when many simplifications are assumed. In previous works [16,28,29], AP has been successfully used to control robots able to perform patient-robot interactions in some Social Assistive Robotics (SAR) [30] applications like CGA or rehabilitation. The systems were tested on real patients with success. However, they were designed using a different PDDL domain model for each task. This solution presents two main problems: (i) all the common elements of the tasks are replicated, so any modification in those parts need to be updated in every domain, and (ii) including new tasks requires to design the domain model from scratch. These limitations can be softened since the tasks share a common structure, which can be extrapolated to other robot-driven interactions in similar health related scenarios.

The contributions of this work are:

1. A conceptual model which generalizes the elements and relations to consider while building robots able of performing different robot-driven HRI tasks sharing a common structure; the robot behavior is assumed to be generated automatically by an automated planner, as described in Section 3.1. The conceptual model supports the organization of the knowledge and its relationship with the execution model.
2. A generic model described as a PDDL domain which formalizes the conceptual model. Given a task, the generic domain together with the corresponding problem, which is specific to the task, allow the planner to generate an execution flow. This execution flow is prepared to deal with corrective actions and exogenous events, as described in Section 3.2.
3. The formalization of four HRI tasks of different nature described in the four PDDL problems, respectively. Three tasks are in the scope of CGA (see Section 2.3) and another one in the scope of rehabilitation (Section 2.4).
4. An evaluation equivalent to the one performed with a robot tested with real patients showing that there is no loss of performance when the robot behavior is defined using the proposed general model.

**Figure 1:**
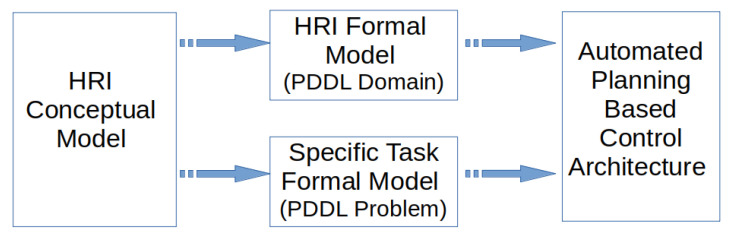
Formal models of HRI for a control architecture based on AP.

The importance of the contributions relies on the fact that the domain of the planning model is generic, and therefore it is shared by the different tasks and defined just once. The domain specifies the rationale of a generic HRI for those tasks. The particularities of each task are defined separately in the problem of the planning model. Once the specific problem is defined, the control architecture uses both, the domain and the problem, to plan the robot behaviour (see Figure 1). This approach overcomes the aforementioned limitations of generating specific models from scratch for different tasks, and greatly facilitates the development of new tasks. To the authors’ knowledge, there are no such general planning models for robot-driven HRI in the literature. All other approaches, either using automated planning or other control techniques, rely on ad-hoc models, created for the HRI task at hand and difficult to generalize even for close related tasks [2,4,5,6].

The next section describes the background of the paper, which includes AP, how it can be used for HRI, and a brief description of the use cases in CGA and robotic rehabilitation. Then, Section 3 introduces the conceptual model representing the knowledge needed to control the interaction. That section describes also a planning domain, based on the conceptual model, which is general for all the considered use cases and extensible to other interaction tasks following a similar structure, as well as an example on how to formalize one of the CGA tasks. An evaluation of the domain model and its ability to guide a robot is presented in Section 4. The paper finishes with conclusions and future work in Section 5.

## 2. Background

This section introduces classical planning, briefly describes an architecture to use it for HRI, and finally it presents the specific use cases that will be modeled in the HRI scope.

### 2.1. Automated Planning

A planning task can be formally defined as a tuple $\Pi =\left\{F,X,A,I,G\right\}$; where *F* is a set of propositional facts, that can only be true or false, *X* is a set of numerical facts, *A* is a set of actions, *I* is the initial state, and *G* are the goals. A state is defined by a subset of propositional facts (those that are true in that state) and by the values of the numerical facts in the state. The goal set *G* is a partially defined state. Each action a∈A is defined in terms of its preconditions and effects, defined both over facts. An action a∈A is applicable in a given state if its preconditions hold in that state. Regarding propositional facts, action preconditions express conditions about the truth value of the corresponding fact. Regarding numerical facts, action preconditions express conditions that include comparison operators (>,<,>=,<=,= ) over their values. Action effects are defined as pairs <c,e> where *c* is the condition triggering that effect and *e* the changes on the state where the action is applied. Effects *e* can be either to add/delete a propositional fact or to modify the value to a numerical fact. Unconditional effects have an empty condition. In this context, a plan $\pi =\left\{a_{1},a_{2},\ldots a_{n}\right\}$ is a sequence of actions such that for each *i* with 1≤i≤n, $a_{i}$ is applicable in $s_{i-1}$, being $s_{0}=I$ and where the goal condition is satisfied in the last state, $G\subseteq s_{n}$.

The former definition corresponds to classical planning with numerical fluents and conditional effects, as defined by PDDL2.1 [26]. This approach allows to achieve a good balance between model complexity and control capabilities. Numerical fluents are useful to represent in a compact way the order of the different interaction elements and phases. Conditional effects are also a way of compacting the representation to be more legible.

In PDDL the models are divided into two files: the domain, which contains the definition of the predicates and numerical variables involved in the states and the action schemes, whose parameters are instantiated on objects in a preprocessing phase of the planner to generate ground actions; and the problem file, which contains the descriptions of the objects in the specific planning task, the initial state of those objects and the goal conditions. Using this partition, a domain file can represent a family of planning tasks, which are specified by different problem files. Any use case for HRI with a general structure that fits the proposed conceptual model can be formalized using the same domain file; different use cases will require different problem files.

### 2.2. Using Automated Planning for HRI

**Figure 2:**
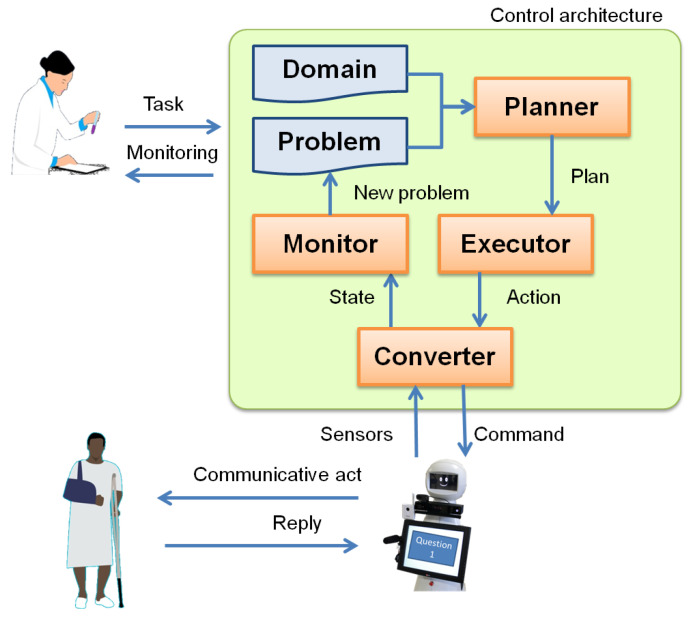
Conceptual schema of the use of Automated Planning for HRI in Social Assistive Robotics.

In addition to a planner that provides the sequence of actions, the use of AP to allow the robot behaving autonomously when conducting an HRI use case in a real environment requires a control architecture [31]. This work assumes one of such architectures is given, specifically the Planning, Execution and Learning Architecture (PELEA) [32]. Its design and implementation are out ofthe scope of this paper, but Figure 2 describes a conceptual schema of how it, or any equivalent architecture, must be used to control a robot in HRI. More technical details on the architecture can be found in the works of Bandera et al. [28] for CGA and González et al. [16] for rehabilitation.

First, the healthcare professional decides the task the robot will perform. The actions it can execute to carry out tasks will be encoded in a generic way in a PDDL domain. The details of the task will be encoded in a PDDL problem. Specifically, the PDDL problem contains the initial state and the goals of the problem to solve. This information allows to instantiate the actions defined in the PDDL domain. Using the domain and the problem, a  *Planner* creates a plan of actions to perform the required task. This plan is forwarded to the *Executor*, which sends the first action to the robot. Planning is usually performed at a high abstraction level, so actions have to be translated into the real low-level commands the robot can execute. This is the duty of the *Converter* module, which also translates the raw data coming from robot sensors (low level information) into a high-level state. The robot then interacts with the patient and receives feedback using its sensors. *Converter* abstracts a high-level state from the information received and sends it to *Monitor*, which checks if the interaction is proceeding as expected. If so, the next action is sent to the robot. If not, the plan is discarded, a new problem reflecting the current state is created and the *Planner* is invoked again to find a new plan. This cycle is repeated until the robot has completed the task.

In the authors’ previous works, a different domain was created for each task (each of the CGA tests and rehabilitation). The approach in this paper goes one step further by defining a general conceptual model that allows to formalize all the four tasks as a unique domain.

### 2.3. Comprehensive Geriatric Assessment Tests

Comprehensive Geriatric Assessment (CGA) [33] is a clinical management strategy for frail elderly patients. It provides a framework for the delivery of interventions to evaluate, on an individual level, the medical, psycho-social, functional and cognitive state of patients, allowing for personalized treatment and follow-up. Usually it is carried out every 6 months and consists of three different steps. The first one is the Clinical Interview, where patients and relatives report their perceptions about patient status. In the second step, Multidimensional Assessments, different tests are done by patients or relatives to evaluate the functional, cognitive, motor and social status of patients. Information gathered during these two first phases is used to create an Individualized Care Plan in step 3.

CGA is a powerful procedure which aims to increase the quality and quantity of life of the patient, but it is also a time-consuming activity challenging the health-care systems capacities. In an attempt to automatize the procedure and save clinicians’ time allowing them to concentrate on more added-value tasks, the European Project ECHORD++ (See http://echord.eu for more information.) launched in 2015 a challenge for the use of robotics to perform phase 2 of CGA. The idea is to have a social robot, the CLARC robot [29], to help health-care professionals in collecting information. The robot should autonomously perform the tests while the health-care professional discusses with the relatives. This may reduce the duration of the CGA process, avoiding waiting time for patient and relatives, and saving clinicians’ time. Examples of the performed tests are:

- The Barthel’s Index Rating Scale [34] measures the functional status asking about patient’s capabilities performing daily living activities. Ten questions with three or four closed-answers are evaluated following a Likert scale structure. Questions like: “Are you able to eat by yourself?”, with possible answers: “Yes, I need no help”, “Yes, but I need some help, for example cutting food”, “No, I need to be fed”. Given that this a closed-answer test, where patient replies verbally or using a tablet, it is relatively easy to perform by a robotic platform.
- Mini-Mental State Examination (MMSE) [35] evaluates cognitive changes in patients suffering from dementia. Orientation, immediate and short-term memory, attention, calculation, recall, language understanding, and ability to follow simple commands are examined. It includes closed-answer questions, but also open-answer ones (“What day is today?”), or even monitoring of simple movements (“close your eyes”) and painting or hand-writing, which poses more challenges from an HRI point of view.
- The Get Up & Go test [36] measures balance and fall risk. Unlike the previous ones it is a pure physical test requiring the patient to stand up from a chair, to walk for a short distance, and to come back to the chair to sit down again. The goal of the robot is both to guide the patient giving verbal instructions, and to place itself in a suitable location allowing to perceive the gait and to evaluate balance and speed.

Clinicians chose these tests because they assess various dimensions of the patient status, are diverse in nature and present different challenges from an HRI point of view. Each of them was initially modeled as a different planning task, i.e., using different domain and problem files for each test.

### 2.4. Robotic Rehabilitation

AP has also been used in an autonomous robot performing non-contact upper-limb rehabilitation therapy for kids with medical conditions like cerebral palsy and obstetric brachial plexus palsy [16,37]. A NAO robot performs a set of predefined arm-poses to be imitated by the patient. In addition it checks the patient’s pose, corrects it in case it does not meet the requirements, and also handles non-expected events as the kid leaving the training area.

Session starts with the patient entering the training room while tracked by the system, which captures body characteristics using a 3D sensor. After greeting the patient, the robot introduces the first exercise; a sequence of poses where each one must be maintained for a certain amount of time. If patient pose is not satisfactory a correction mechanism is started: After the first failed attempt, the robot points the incorrect arm and asks the kid to correct it. If the pose is still incorrect, the robot imitates the wrong posture and shows how to reach the correct one, in what is called “mirrored correction”. After these two tries, if the patient keeps failing the pose is skipped (An example of a rehabilitation session can be seen at: https://www.youtube.com/watch?v=75xb39Q8QEg).

## 3. Design of a General Domain Model

Based on the authors’ experience in the previous SAR projects a generic model of human-robot interaction controlled by AP that can be used in similar scenarios has been developed. This section presents a conceptual model that captures all the types of interactions that were detected from previous works. Using this conceptual model a unique PDDL domain able to control a robot while performing different tasks has been designed. The domain file is unique for all the tasks; all the specific information is included only in the problem file. In this sense, the domain file formalizes the structure of the human robot interaction while the problem files formalize the specific task, session and patient information.

The interaction is driven by the robot. It sends petitions to the human to perform some task as, for example, answering a question. Then, in the best case, the recipient will perform the task and the robot will continue to the following one. The model proposed in this section is adequate for interactions of this type, with a common structure where the interaction is divided into several sequential phases.

The next subsection defines a conceptual model that describes the patient-robot interactions. Using this model allowed us to create a general planning domain useful for generating plans for controlling the behavior of the robot for different human-robot interaction tasks, which is presented in Section 3.2. Currently, it has been tested for CGA tests and the rehabilitation tasks described above. The obtained planning domain is the same for the different tasks. However, each planning problem defines the particularities of every task and it is therefore specific to it.

### 3.1. Conceptual High Level Knowledge Model

**Figure 3:**
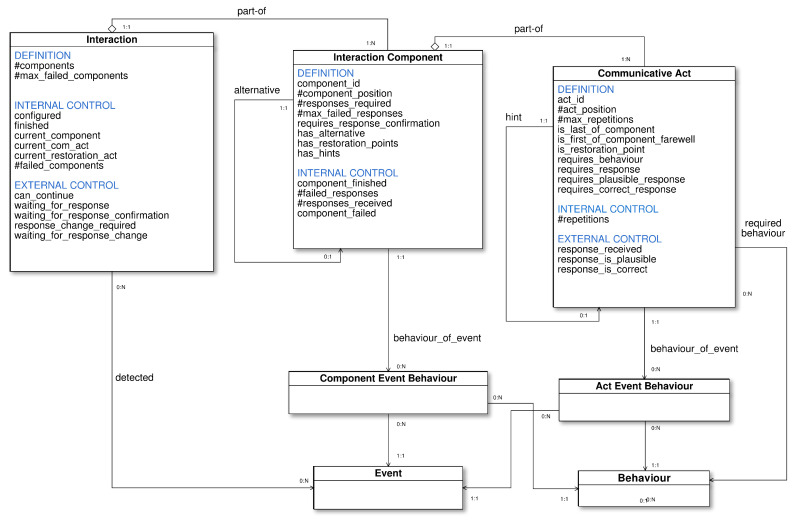
High level interaction conceptual knowledge model.

The planning domain is based on the conceptual knowledge model summarized in Figure 3. It represents, in a generic way, the main concepts to allow the high level definition and management of the interaction between the robot and the patient.

Each of the interaction phases [38,39] is called *interaction component*. In CGA tests, each one of these components is a part of the test: the test introduction, the first question, the second question, etc. Each component is associated several ordered *communicative acts*. A communicative act represents an uninterrupted act of the robot to communicate something to the recipient. It is typically a sentence expressed either by voice or text. It can also involve some additional behavior of the robot as showing a video, adopting a particular pose, etc.

Two broad types of communicative acts are considered: directive and non-directive. Directive acts [40] imply asking, ordering, etc., so a response of the recipient is expected immediately after them. The response will be typically a spoken answer but it could be also to do something. Such a response is not required by non-directive acts. Examples are acts to give some information as the test introduction, the introduction at the beginning of a question, etc.

Following the defined interaction model, the proposed conceptual model includes three main concepts: *Interaction*, *Interaction Component* and *Communicative Act*. The *Interaction* concept represents the overall interaction which is composed of a sequence of *Interaction Components*. At the same time, every *Interaction Component* is composed of a sequence of *Communicative Acts*. There are also other concepts related to the definition and management of the different events that can occur during the interaction and interrupt the nominal execution flow. In those cases, the robot must perform some corrective behavior accordingly to the detected situation. The involved concepts are *Event* and *Behavior*. The former represents the possible events that can be detected. The later represents the possible behaviors that the robot can perform. The concepts *Component Event Behavior* and *Act Event Behavior* allow to relate events and behaviors to components and communicative acts respectively. They define which should be the behavior when an event is detected during a component or after a communicative act. Additionally, communicative acts can be also associated to a behavior to be executed during the act, as for instance showing a video.

The conceptual model is the base for defining a planning domain for controlling the high level behavior of the robot. The information that comes (or can be modified) from the environment through the robot sensors is considered as *external* to the planner. Thus, the conceptual model in Figure 3 distinguishes between three types of attributes:

- **Definition**: those that define the structure and characteristics of the interaction.
- **Internal**: those that express internal control information about the current state. These attributes are never modified externally.
- **External**: those that represent control information that could be modified externally, i.e., perceived by sensors.

Table 1, Table 2 and Table 3 contain a detailed description of every attribute in the model. The symbol *#* refers to numeric attributes. Attributes without this symbol are either identifiers or *Boolean*. The remaining of this section explains the most relevant parts of the model.

Communicative acts are identified just by labels. The specific texts of the sentences together with their corresponding labels are stored at the low level. When a response is expected just after the robot performs a communicative act, it should be defined as a *required_response* act.

The recipient responses associated to communicative acts are not explicitly represented in the conceptual model, since they are processed at a lower level. However, the high level control could be different if the response is or not received. This is represented by the attribute *response_received*. Also, there could be cases where it is necessary to validate that the response is plausible and/or correct (attributes *requires_plausible_response* and *requires_correct_response*). In a question, plausible responses are those that answer the question meaningfully but are not necessarily correct. For instance, the question *What weekday is today?* has seven plausible answers, but only one of those is correct. The high level plans will contain response-related actions such as *validate-plausibility* or *validate-accuracy*, to trigger validations of the response in the low level. The result of the corresponding validation is represented by the attributes *response_is_plausible* and *response_is_correct*.

Additionally, a communicative act can be repeated several times. This is useful to ask again when the recipient does not provide a response. Every communicative act is associated a maximum number of repetitions, *#max_repetitions*, indicating the maximum number of times the act can be repeated. In the low level, the same communicative act label can be associated to several sentences to express the same in different ways for different repetitions, improving the usability of the system.

The model includes also the possibility to include a hint into a communicative act. A hint is another communicative act related to it. This is useful to help the recipient when she/he provides a plausible but not correct response. This is represented by the relation *hint*.

Finally, some communicative acts can be marked as restoration points, attribute *is_restoration_point*. Restoration points define points of the interaction to come back when some action is interrupted before the next restoration point is reached, to recover the interaction context [27]. For example if the patient leaves the room while the robot is waiting for an answer, when the interaction is resumed the execution should start by asking the question again.

Components of the interaction domain, as test questions, can involve several directive communicative acts. Therefore, they can have either several responses or several alternative ways for obtaining the response. If there is no response for a directive communicative act (or the response is not adequate), it is considered as a failed act (attribute *component_failed*). There is a maximum number of failed responses for every component (*#max_failed_responses*). When that number is exceeded the component is considered as a failed component. The interaction control keeps track of the number of failed components since this number can be relevant to decide what to do next.

It is also possible to define an alternative component, represented by the relation *alternative*. The idea is to offer another chance to obtain the responses with a different but equivalent component when the first one fails.

All unpredictable information that could interrupt the nominal execution is summarized into the model by the *can_continue* attribute of the *Interaction* concept, that depends on external information. Examples of unpredictable events that can interrupt the execution can be that the recipient is absent (the robot sensors do not locate the person) or the robot battery is very low.

### 3.2. Formalization of the Conceptual Model Using Classical Automated Planning

The conceptual model presented in the previous section constitutes a framework specifying the actions to perform and the information to consider by a robot driving a family of HRI tasks in social assistive robotics. But to be used in practice the conceptual model needs to be grounded in a real model used by a deliberation technique [31]. In this work the model is formalized using Automated Planning, which means the conceptual model needs to be formalized in a domain and a problem. Thanks to the conceptual model the domain will be unique for the family of HRI tasks; the details of each one, including possible adaptations to the patient conditions, will be modeled using different problems. The methodology followed to create the domain, including the selection of the planning approach, the planning horizon, the model partition, or the design of the actions and the states, is presented in a previous work [27]. To summarize, since it is a single-robot environment there is neither need for concurrent actions nor for complex time reasoning. Then, the model was created using classical planning, and  desfined using PDDL2.1 [26]. The planning horizon of the domain is the full interaction, either a CGA test or a rehabilitation session. Uncertainty in actions and external events have been handled by establishing a *nominal flow*. It describes a seamless interaction between the robot and the patient where both behave as expected. If the interaction diverges from the nominal flow or an unexpected event appears, replanning is used to perform some corrective actions that bring the flow back to the nominal one. This model is independent of the planning architecture used: the only assumption is that it accepts PDDL 2.1 and that a planning-replanning approach like the one presented in Figure 2 is followed. In that sense other architectures like ROSPlan [25] can be used.

In PDDL domain actions are defined by enumerating their preconditions and effects. Both, preconditions and effects, refer basically to information defined in the conceptual knowledge model which has been formalized using predicates and functions. This section provides a general explanation of the domain actions. The planning domain contains two kinds of actions:

- Actions belonging to the nominal flow of execution of an interaction. The nominal flow represents the case where the interaction advances in the best possible way; and
- Corrective actions, that try to return the execution to its nominal flow when some event considered as unexpected occurs.

**Figure 4:**
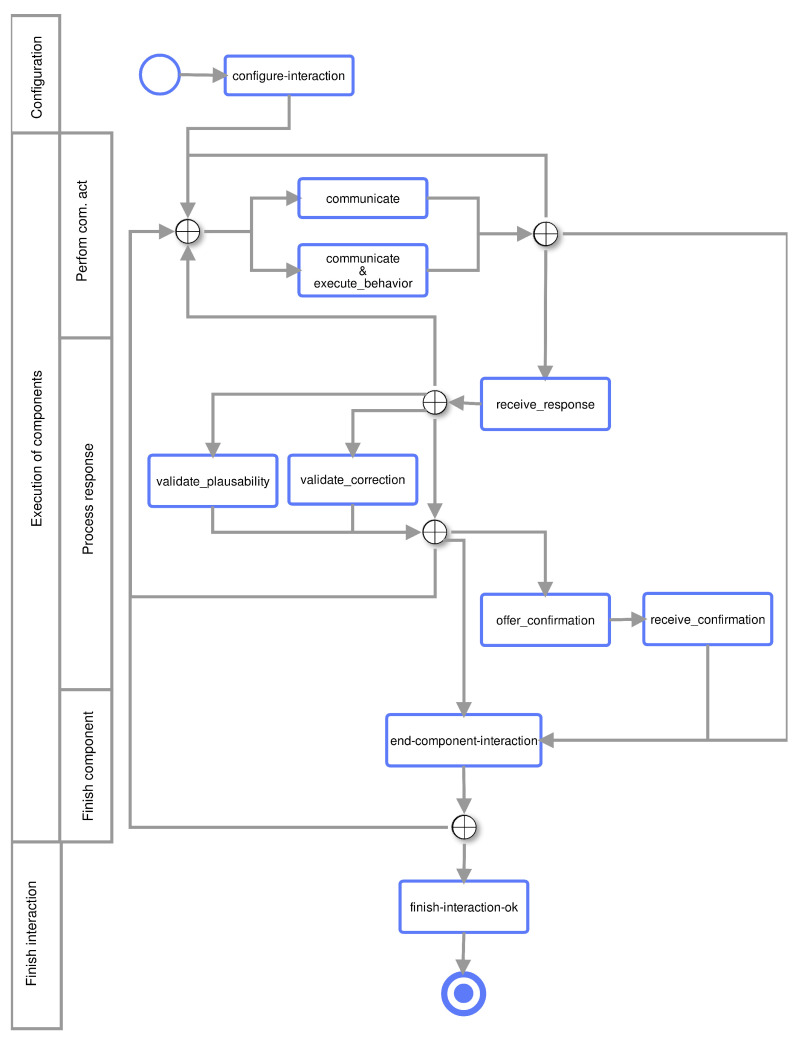
Nominal flow interaction diagram.

Figure 4 shows a diagram with the actions of a possible nominal execution flow. The nominal flow depends on the specific interaction definition. This diagram includes only some of the possible cases. It contains bifurcations depending on that specific definition. The nominal execution flow is divided into three broad phases: configuration, execution of components and finish interaction. The execution has three steps for every component: perform communicative act, process answer and finish component. The first two steps can be repeated depending on the communicative acts of the component.

The configuration phase contains the action *configure_interaction*. The configuration is performed at the low level, so that the high level just controls it has been done. The configuration consists of the selection of the interaction language, the verbal tense, etc. Then, the nominal execution flow continues as follows:

1. The robot performs the first communicative act of the current component. It may involve to execute a behavior, as for instance showing a video. Involved domain actions are *communicate* or *communicate&execute-behavior*.
2. The robot keeps doing 1. until all communicative acts of the component have been executed or a response is needed (i.e., the communicative act requires a response). In that case, it executes the action *receive_response*.
3. Then, the robot waits for the response, giving the user a maximum number of attempts to receive it. Each attempt involves to repeat the last communicative act to ask again. Some atomic acts may require to validate the answer plausibility (for instance, that it is included in the set of valid answers for a closed-answer question) or to validate its accuracy (for instance, that the answer makes sense for an open-answer question). If the answer is not acceptable, the communicative act is repeated to its maximum number of attempts.
4. If the maximum number of attempts is reached, the flow continues to the following communicative act which was defined as a different way of achieving the answer. If no such way exists the nominal flow is interrupted.
5. If the response is obtained, it could be the case that it belongs to a component that requires a response confirmation. In this case, the nominal flow will contain two actions: *offer_confirmation* and *receive_confirmation*. When the confirmation is not received the nominal execution flow is interrupted.
6. The final action is *end-component-interaction*, which allows either going to the next question or to finish the interaction.

In addition to the actions described in the nominal execution flow, the domain contains the control action *prepare-component-farewell*. It is not associated to any robot action, but modifies the control to advance to the first communicative act of the component farewell. This action can appear in the nominal control flow for components whose communicative acts define alternative ways of getting the response, when the answer is received before the last of those alternative ways.

The domain contains corrective actions to deal with the following situations:

- Change of response. It is produced when a response should be confirmed by the patient and he/she does not confirm it. There are two corrective actions to solve this situation: *offer-response-change* and *receive-changed-response*.
- Hint should be activated. It is produced when it is required to provide a hint to the recipient given that her/his response is not plausible or it is not correct. Associated actions are *validate-plausibility-and-hint* and *validate-correction-and-hint*.
- Response failed event. It is produced when a communicative act for obtaining the answer has been repeated its maximum number of times and the answer (either a plausible or correct one) has not been received. The involved corrective actions are: *raise-response-failed-event* and *handle-wrong-response*. The second one forces the low level to execute the behavior defined to manage an event called *response-failed-event* and to advance to the next communicative act.
- Component failed event. It is produced when the maximum number of failed answers for a component has been reached. In such case, the corrective actions are *raise-component-failed- event-no-response*, *raise-component-failed-event-max-repetitions* and *skip-component*. The action *skip-component* can be activated also directly by an event induced by the patient. This action advances to the next component and annotates the interaction for calling the supervisor (doctor) at the end. When the component has an alternative, the action *switch-to-alternative-component* is used to change the execution flow to the alternative before skipping it.
- Maximum number of failed components event. It is produced when the maximum number of failed components has been reached. The corrective action is *raise-max-component-failed-event*. This action marks as detected an event defined to identify that situation.
- Detected event. It is produced when an event has been detected during the execution of a component. It forces the low level to execute the behavior defined to manage the corresponding detected event. There are two corrective actions in this case: *handle-event-and-resume* and *handle-event-and-go-back*. The first one is applied when the interaction can continue from the current point once restored the situation generated by the event. The second one is applied when it is necessary to go back to a restoration point.
- The interaction requires to call the supervisor at the end. It occurs if there has been a situation during the interaction that requires the supervisor to be called and the end. The involved action is: *finish-interaction-call-supervisor*.

This domain has enough expressive power to guide the interaction for the Barthel, Minimental and Get up & Go tests and also for upper-limb rehabilitation tasks, included in the evaluation of the next section. The files can be downloaded from a public repository (https://bitbucket.org/fjaviergp/planningforhri).

**Figure 5:**
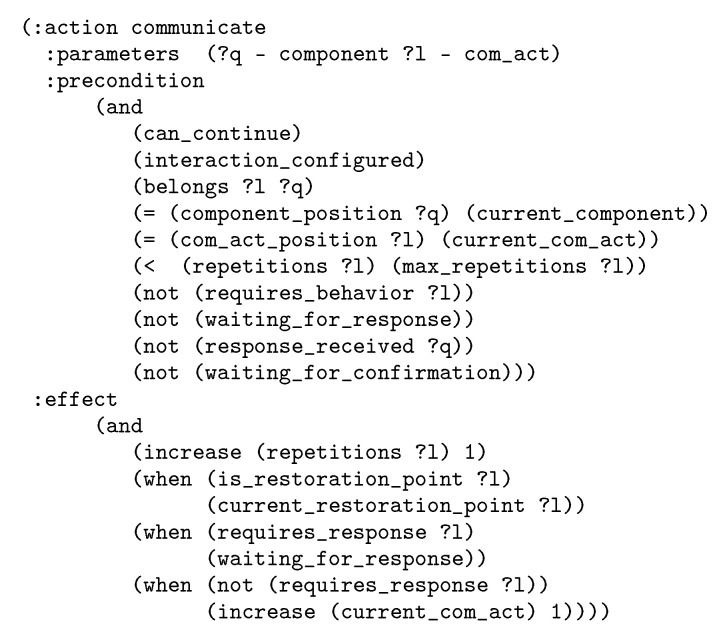
Example of domain action.

The general domain contains 25 actions. Figure 5 shows one of them, to illustrate the actions’ structure. This action will appear in plans to indicate that the robot has to perform a communicative act of a component that does not require to execute a behavior. As it can be observed the action preconditions control that nothing is interrupting the interaction and that the interaction has been configured. Also, they control that the communicative act corresponds to the current interaction state and that it was not repeated more times than the maximum allowed repetitions. Then, they contain some checks about the response, as for instance that it has not been received. The action effects increase the number of repetitions of the communicative act. There are also some conditional effects related to the response control and to the update of the current restoration point.

**Figure 6:**
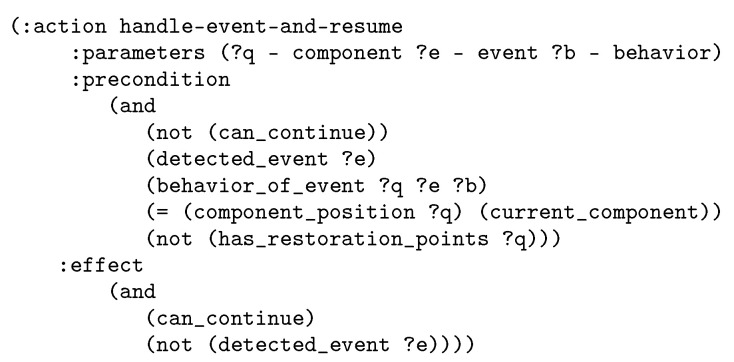
Example of domain action to recover from an interruption of the nominal flow.

The action communicate belongs to the nominal flow of the interaction. As aforementioned, the domain contains alsocorrective actions, as the one showed in Figure 6. The execution of this specific action forces the low level to execute the behavior defined to manage the corresponding detected event. The action is for the case that the interaction can continue from the current point once restored from the situation.

### 3.3. Formalization of the Different Tasks in PDDL Problems

**Figure 7:**
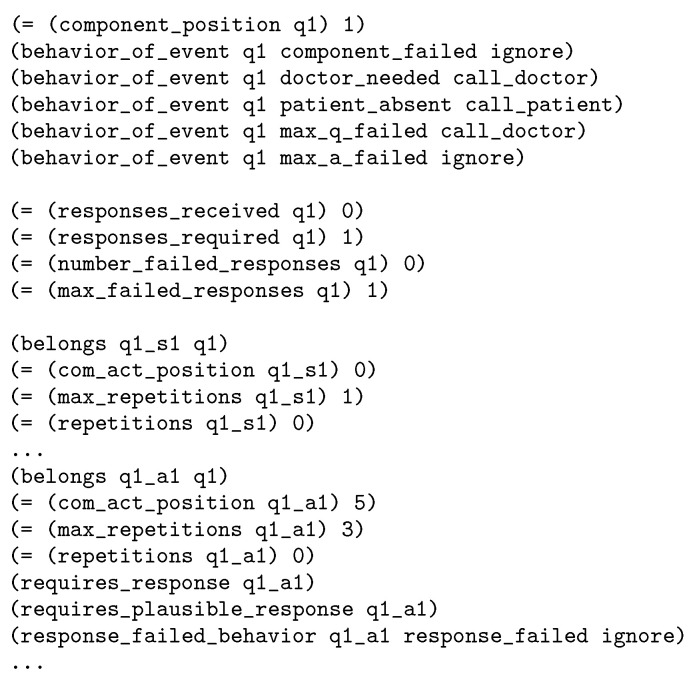
Fragment of problem definition for Bathel test. First question (component q1).

As described above, the PDDL domain is shared by the different HRI tasks to be addressed, but there are different planning problems defined for each of the use cases defined in Section 2.3 and Section 2.4. As an example, Figure 7 shows a fragment of the problem definition for the Barthel test. Specifically, it shows part of the definition of the first component, which represents the first question of the test (identifier q1). The definition starts indicating the question position and defining the behaviors for different events. This question is composed of several communicative acts, with identifiers q1_<X>. Each communicative act has it own definition indicating its position, the maximum number of repetitions and some other information, as for instance that the act q1_a1 requires a plausible response.

**Figure 8:**
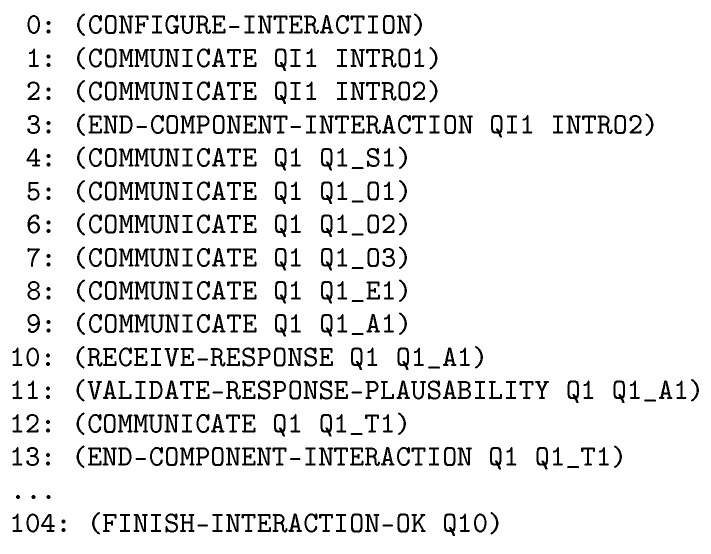
Plan fragment of the nominal plan for the Barthel test.

Finally, and also as illustration, Figure 8 shows part of the nominal plan for the Barthel test. The plan contains the high level actions the robot has to execute. The Barthel test is composed of 10 questions (from q1 to q10). The complete plan in this case contains 104 actions.

## 4. Evaluation

This section presents the experimental results collected from the use of the general planning model for the four proposed HRI tasks. Section 4.1 introduces the experimental settings and scope, and Section 4.2 shows the particular results of the evaluation.

### 4.1. Experimental Settings and Scope

In previous works [29,37] four different PDDL models were created (four domains and four problems) for the four applications shown before and tested them with real patients. Having four different domain files and fine-tuning them as a result of evaluations turned out to be a time-consuming and error-prone process. Lessons learned after those evaluations gave rise to the general model introduced in this paper, which systematizes the interactions using a common conceptual model. This section shows the evaluation of the unified model and its comparison to the previous specific ones. Evaluation is done in two dimensions: On the one hand the domain and problems created must allow finding a plan respecting the real-time constrains imposed by patient-robot interaction. On the other hand, the plan created must be able to control the robot while performing both CGA tests and rehabilitation sessions in a similar way as the specific domains allowed. It is important to bear in mind that building specific models for each particular HRI task is the common approach found in the state-of-the-art [4,5,6], so the aim of the proposed evaluation is to check if that specific domains can be replaced by a single one keeping reaction times and making the robot to externally behave in the same way.

The experiments in this section are defined using the same framework as in the experiments with real patients: the PELEA [16] planning-execution architecture, and the Metric-FF [41] high-level planner. The experiments were run in a Linux 64 bits Intel Xeon 2.93 GHZ Quad Core processor with 2GB RAM. Given that pilot studies with real patients have ended, the results here correspond to simulations with the following settings. There is a 10% probability that the robot loses track the patient. Also, for Barthel and Minimental tests, an interaction error has been included, with a 30% probability, simulating that the patient does not respond or the robot does not receive the answer. In the case of Get up & Go test, several detection errors have been included: the patient is not detected near the chair (10%), the patient is not detected seated (20%), and the robot detects the patient walked more than the required distance (10%). Finally, in rehabilitation two different exercises were simulated including an error of 20% in the poses performed by the patient.

### 4.2. Results

This section shows the results of the proposed evaluation. In particular, each row in Table 4 provides the results for a given test using the general domain, and also the results of the ad-hoc formalizations, i.e., the old PDDL domain and problem files created specifically to solve the corresponding application. All these PDDL files are also public and can be downloaded from the repository (https://bitbucket.org/fjaviergp/planningforhri).

In particular Table 4 shows the number of actions in the executed plan, the number of replanning episodes needed due to an unexpected event, the number of seconds needed to find a plan, and the average time the system needs to send the next action to the robot in milliseconds (*Response*). The table shows means and standard deviations from ten different executions for each domain and test.

The first aspect that is important to notice is that the general domain creates longer plans. Therefore, it allows to improve the generalization capacity, but at the cost of a higher number of actions. However, these additional actions just control the internal flow of the planning process and, hence, there are no associated low-level actions the robot needs to perform. It must be remembered that plans created using both the specific and general domains are low-level equivalent, so the actions performed by the robot are similar, there is no external difference on robot behavior by using one or other approach. As an example, in the CGA tests the corrective action *handle-wrong-answer* has no any associated behavior to manage this event as nothing must be done if an answer is not correct and, hence, it is ignored.

A second aspect to notice in Table 4 is that general plans are found faster in the general domain than in the specific ones. This is because one of the aims of the general domain is to simplify the previous ones. It contains only the required information to reason, leaving other aspects to the low level. For example, pauses between speech segments are not planned in the general domain but left to the low-level, while they were planned in the original ones. This will make the general domain faster and easier to understand.

The third aspect to consider from Table 4 is that the number of replanning situations (dimension Replan in Table 4) is not affected by the use of one or other domain and, in both cases, the mean response time per action of PELEA (dimension Response) is similar. Response times allow for a correct social interaction.

In summary, the proposed general planning model can be used to perform a wide variety of HRI tasks, without losing performance against specific planning models, and even improving planning times. In fact, this is the main benefit of the proposed approach in comparison to the previous work: a general classical planning model that can be easily adapted to automatically conduct different HRI tasks, avoiding fine-tuning of specific planning models and their time-consuming and error-prone modifications throughout the evaluations.

## 5. Conclusions

This paper introduced a conceptual model and a planning domain for the formalization of the interaction control for a social robot. The planning domain was defined in PDDL, the standard language for automated planning, so any domain-independent planner can be used to generate plans to control the robot. Four use cases were developed in different areas related to healthcare: CGA tests and rehabilitation sessions.

Human-robot interaction, and specifically the interaction in the defined use cases, is a stochastic task which, a priory, does not seem to be suitable for a classical planning approach. However modeling the task accurately, and supported by a complete planning, execution and monitoring architecture [28] achieves a reasonable trade-off between the model construction cost, the real time requirements, and the complexity of robot behavior. The main contribution on this aspect is the idea of nominal flow of interaction plus corrective actions, which allows to handle the inherent uncertainty appearing when working with humans in real environments, while maintaining the models simple and the reaction times low.

The approach presented in this paper showed that different interaction tasks do not necessarily require different formalization of the planning domain but that a general domain can be used so, when new tasks are required, only the problem file should be changed, which permits a better long-term maintenance and extension of the whole system. This is the main advantage of using a conceptual model and generic domain: their ability to represent different types of HRI and to produce plans in real time. Such ability for generalization represents a strong contribution over previous state of the art, in which the behavior of the HRI is modeled ad-hoc, in most of the cases as FSMs, attending to the specific needs of the task, and without the ability that their specific solutions can be reused for other HRI tasks.

The introduced conceptual model is designed for time-limited interactions between the robot and the patient, where the goal of the robot is the patient to do something (answering a test, doing some exercises, etc.). It does not consider long-term interactions as the ones shown by other works [14]. This approach is particularly suitable for applications where a nominal behavior of the robot can be defined, considering any event deviating from this nominal flow as a disruption that needs to be solved by means of corrective actions. The planning/replanning approach could be also used in domains where no such expected flow can be specified or where it is more likely it will be interrupted, by either increasing the number of considered events, the number of corrective actions or the replanning episodes. But in those scenarios other approaches as timeline-based planning [14], probabilistic planning [42] or Markov Decision Processes could be more appropriate.

The experimental evaluations show that the number of actions of the general model is higher than the obtained by specific models, i.e., the proposed general domain allows to model a wide variety of HRI tasks, but it also requires a higher number of actions to solve each particular task. However, most of these additional actions are control actions that do not impact in the time required to solve each task. Therefore, the real-time planning requirements of the platform are satisfied, and plans are generated in less than one second even in cases where replanning episodes are required.

Possible future works include the modelling of new tasks at the request of clinicians, increasing the number of procedures that can be automatized with the system and the formalization of some other different types of questions that can appear in CGA tests. This formalization and the use of a shared single domain is a key component for a graphical user interface, currently under development, that will allow to build models almost without technical support or knowledge of PDDL [43].

Another future line in the hope that the built CGA and rehabilitation robots keep being used with real patients in real interactions, is to learn from these interactions to better tailor their behavior to each person. From the modeling point of view the nominal flow can be adapted to each patient, even if taking into account that it is a medical procedure with strict requirements and little space for changes. Aspects like the preferred interaction means or the probability of failing in a given interaction will be used to adapt the system to each user. The experience gathered in real evaluations will allow also to extend the number of unexpected events and of corrective actions. Here, works like the one of Konidaris et al. [44] seem a good starting point.

## Figures and Tables

**Table 1:** Description of definition attributes.

Concept	Attribute	Description
Interaction	*#components*	Number of components
	*#max_failed_components*	Max number of allowed failed components
Component	*component_id*	Label to identify the component
	*#component_position*	Position of the component in the interaction
	*#responses_required*	Number of recipient responses required for the component
	*#max_failed_responses*	Max number of failed responses allowed
	*requires_confirmation*	Before finishing the execution of the component the user must confirm the information is correct
	*has_alternative*	There is an alternative component
	*has_restoration_points*	The component has restoration points in its com. acts, for going back if necessary
	*has_hints*	One or more of the com. acts of the component has a hint
Com. Act	*act_id*	Label to identify the com. act
	*#act_position*	Position of the act in the corresponding component
	*#max_repetitions*	Maximum number of times it can be repeated
	*is_last_of_component*	This act is the last one of the component
	*is_1st_component_farewell*	This act is the first one of the component farewell
	*is_restoration_point*	It is a restoration point for going back if necessary
	*requires_behavior*	Behavior that should be executed during the act
	*requires_response*	This act is a directive one. A response is required
	*requires_plausible_response*	The response after this act should be validated
	*requires_correct_response*	The response after this act should be correct

**Table 2:** Description of internal attributes.

Concept	Attribute	Description
Interaction	*configured*	The interaction has been configured
	*finished*	The interaction is finished
	*current_component*	Id. of current component
	*current_act*	Id. of current com. act
	*current_restoration_point*	Id. of the com. act representing the current restoration point
	*#failed_component*	Number of failed components
Component	*component_finished*	This component is finished
	*#failed_responses*	Number of failed attempts to achieve the response
	*#responses_received*	Number of responses received for this component
	*component_failed*	This component is considered as failed

**Table 3:** Description of external attributes.

Concept	Attribute	Description
Interaction	*can_continue*	The test can continue. This predicate is removed when the interaction cannot continue due to several causes as low battery, the patient asked for help or the stop button has been pressed
	*waiting_for_response*	The recipient should give a response now
	*waiting_for_confirmation*	The recipient should confirm the response now
	*response_change_required*	The recipient has requested a response change
	*waiting_for_response_change*	The recipient should change the response now
Com. Act	*response_received*	The response has been received
	*response_is_plausible*	Plausibility of response has been checked
	*response_is_correct*	Correction of response has been checked

**Table 4:** Comparison of the general and specific domains in Barthel, Get up & Go, Minimental and Rehabilitation.

Test	Domain	Actions	Replan	Planning (s.)	Response (ms.)
Barthel	General	123.5±8.4	6.3±2.8	0.08±0.01	281.5±5.3
	Specific	102.0±5.3	5.8±3.3	0.51±0.01	277.7±6.8
GetUpGo	General	33.0±2.7	3.0±0.9	0.01±0.0	232.5±4.9
	Specific	20.0±1.8	2.2±0.7	0.05±0.02	236.1±5.6
Minimental	General	178.2±6.3	8.4±3.4	0.18±0.0	284.8±7.9
	Specific	160.4±5.2	9.2±4.7	1.53±0.02	281.1±5.7
Rehabilitation	General	75.2±6.7	4.2±0.9	0.01±0.0	242.5±5.8
	Specific	58.4±7.8	5.2±1.7	0.02±0.0	246.2±7.6

## Abbreviations

The following abbreviations are used in this manuscript:

AP	Automated Planning
CGA	Comprehensive Geriatric Assessment
HRI	Human-Robot Interaction
FSM	Finite-State machine
MMSE	Mini-Mental State Examination
PDDL	Planning Domain Definition Language
PELEA	Planning, Execution and Learning Architecture
SAR	Social Assistive Robotics
